# No supra-additive effects of goserelin and radiotherapy on clonogenic survival of prostate carcinoma cells *in vitro*

**DOI:** 10.1186/1748-717X-2-31

**Published:** 2007-08-26

**Authors:** Robert M Hermann, Dag Schwarten, Stefanie Fister, Carsten Grundker, Margret Rave-Frank, Mirko Nitsche, Andrea Hille, Paul Thelen, Heinz Schmidberger, Hans Christiansen

**Affiliations:** 1 Department of Radiotherapy, University hospital, Robert-Koch-Str. 40, 37075 Göttingen, Germany; 2 Department of Gynecology, University hospital Göttingen, Robert-Koch-Str. 40, 37075 Göttingen, Germany; 3 Department of Urology, University hospital Göttingen, Robert-Koch-Str. 40, 37075 Göttingen, Germany; 4 Department of Radiotherapy, University hospital, Langenbeckstr. 1, 55131 Mainz, Germany

## Abstract

**Background:**

Oncological results of radiotherapy for locally advanced prostate cancer (PC) are significantly improved by simultaneous application of LHRH analoga (e.g. goserelin). As 85% of PC express LHRH receptors, we investigated the interaction of goserelin incubation with radiotherapy under androgen-deprived conditions in vitro.

**Methods:**

LNCaP and PC-3 cells were stained for LHRH receptors. Downstream the LHRH receptor, changes in protein expression of c-fos, phosphorylated p38 and phosphorylated ERK1/2 were analyzed by means of Western blotting after incubation with goserelin and irradiation with 4 Gy. Both cell lines were incubated with different concentrations of goserelin in hormone-free medium. 12 h later cells were irradiated (0 – 4 Gy) and after 12 h goserelin was withdrawn. Endpoints were clonogenic survival and cell viability (12 h, 36 h and 60 h after irradiation).

**Results:**

Both tested cell lines expressed LHRH-receptors. Changes in protein expression demonstrated the functional activity of goserelin in the tested cell lines. Neither in LNCaP nor in PC-3 any significant effects of additional goserelin incubation on clonogenic survival or cell viability for all tested concentrations in comparison to radiation alone were seen.

**Conclusion:**

The clinically observed increase in tumor control after combination of goserelin with radiotherapy in PC cannot be attributed to an increase in radiosensitivity of PC cells by goserelin in vitro.

## Background

Luteinising hormone releasing hormone (LHRH) analoga play an important role in the treatment of prostate carcinoma (PC). As an alternative to surgical castration to suppress testosterone levels they are used in the palliative treatment of advanced disease. Furthermore, in locally advanced disease they improve overall survival when given simultaneously to curative radiotherapy [1-3].

The mechanism of this enhancement of survival is still obscure. Interestingly, several older trials that compared radiotherapy with radiotherapy and surgical castration or estrogen application did not show an improvement in survival [4-7] except one [8]. This might be due to the non-randomized study design of several trials and different clinical endpoints.

*In vitro *studies that investigated oncological relevant endpoints like clonogenic survival could not demonstrate an enhanced radiosensitivity of PC-cell lines by testosterone ablation [9]. *In vivo *significant tumor regrowth delay was seen after androgen ablation [10-14]. Two mechanisms for the clinically observed improvement in overall survival after combination therapy were postulated: a) an additive cell killing between androgen ablation and radiotherapy and b) reduced tumor regrowth kinetics after androgen ablation [14]. Interestingly, about 85% of PC express LHRH receptors [15]. The stimulation of these receptors reduces via interferences with the epidermal growth factor (EGF) receptor system the proliferation of PC-cells *in vitro *and *in vivo *[16-18]. This is why we postulated that the clinically observed improvement in overall survival by the combination between LHRH agonists and radiotherapy might be explained by an increased radiosensitivity of PC-cells after LHRH agonist exposure. To our knowledge, *in vitro *studies testing this hypothesis have not been published yet. LNCaP and PC-3 cells were stained for LHRH receptor expression and analyzed for effects of goserelin incubation on protein expression and phosphorylation. Clonogenic cell survival and cell viability were measured after incubation with different goserelin concentrations and radiation doses.

## Methods

### Cell lines and cultures

The PC cell line LNCaP (ATCC nr. CRL1740) was chosen as an androgen-responsive model of PC, the cell line PC-3 (ATCC nr. CRL1435) as an androgen-independent system.

Cells were cultured in Dulbecco's minimal essential medium (DMEM, Invitrogen, nr. 41965-039, Paisley, Scotland) supplemented with 2% glutamine, 1% sodium pyruvate (all purchased from Sigma, Steinheim, Germany), 1% penicillin and streptomycin (Biochrom, Berlin, Germany]) and 10% inactivated fetal bovine serum (Biochrom, nr. S0115) in 10% CO_2 _atmosphere. To test selectively the effects of goserelin without any other hormonal stimuli cells were grown in "hormone free medium" (HFM): phenol red free DMEM (Sigma, nr. D2902) supplemented with 4.5 g/l glucose (Sigma), 2% glutamine, 1% sodium pyruvate, 1% penicillin + streptomycin and 10% charcoal-stripped fetal bovine serum (Biochrom, nr. S3113).

### Staining for LHRH

Staining was done following a protocol as published previously [19]. 10.000 cells were seeded in each well of an 8-chamber slide. 24 h later the cells were washed in PBS, incubated with 1 mol/l glycine for30 min, washed in PBST (0.2% BSA, 0.1% Triton X-100 in PBS) for 2 × 15 min, and treated in PBSTN (5% FBS in PBST) for 10 min. The first antibody was a monoclonal mouse anti-human LHRH receptor (clone A9E4; Research Diagnostics, Flanders, New Jersey, USA), diluted 1:20 in PBSTN; the cells were incubated therein at 4°C overnight. After three washes in PBST, the cells were incubated with PBSTN for 30 min and then treated with the Histostain SP kit for mouse primary antibody (Zymed, San Francisco, California, USA) according the manufacturer's instructions. Dako (Carpinteria, California, USA) 3,3'-diaminobenzidine liquid substrate-chromogen system was used as substrate. Controls were performed by omission of the primary antibody.

### Cell viability assay

Goserelin acetate was kindly provided from Astra-Zeneca (Wedel, Germany). It was dissolved in H_2_O stock solution. Exponentially growing cells in maintenance cultures were washed twice with PBS and incubated with HFM. 24 h later the cells were detached with trypsin/EDTA, counted and diluted. 1000 cells were transferred into each well of a 96-multiwell plate in HFM containing different concentration of goserelin (0.001 – 10 μM or water control). 12 h later the plates were irradiated with 0 – 4 Gy using a linear accelerator (Varian, Palo Alto, USA) with 6 MV (dose rate of 2.4 Gy/min). 12 h, 36 h and 60 h later cell viability was determined using the CellTiter-BlueTM assay (Promega, nr. G8081, Madison, USA) according the manufacturer's instructions. The wells were incubated for 4 h with 20 μl of CTB-reagent, absorption was measured in a photometer at 570 nm and 620 nm (reference). This assay measures the metabolic capacity of cells using the reduction of resazurin. In preliminary tests the absorbance shift was proportional to the number of seeded tumor cells.

Experiments were performed in triplicate and repeated at least three times. The results were normalized to the specific irradiation controls (without goserelin) to demonstrate synergistic effects between goserelin and irradiation.

### Colony forming assay

Cell survival was evaluated using a standard colony-forming assay. For LNCaP 1500 – 5000 cells were plated per 25 cm^2 ^flasks for low to high doses of radiation (0 Gy, 0.5, 1, 2, 4). Two days later the flasks were washed with PBS and cells were incubated with HFM to exclude any other than the studied hormonal effects. After 24 h goserelin (0.01 μM and 10 μM) was added. 12 h later the flasks were irradiated with 6 MV. 12 h later the cells were washed and incubated in normal culture DMEM without goserelin. After more than 6 doublings (at least 15 days, change of medium every 7 days) the experiments were stopped. The cell layer was fixed with 70% ethanol and stained with crystal violet. Scoring was done under a microscope. Colonies with more than 50 cells were counted as survivors.

As PC-3 cells proliferated much faster than LNCaP, the strategy had to be adapted for this cell line. Washing and incubation with HFM was done in the maintenance cultures. After 24 h cells were detached using trypsin/EDTA, counted, diluted and replated in 25 cm^2 ^flasks in FMH + goserelin. 12 h later the flasks were irradiated. Experiments were performed in quadruplicate and repeated at least three times.

### Protein extraction and Western Blot analysis

Cells were grown to 80% confluence in 75 cm^2 ^culture flasks. The flasks were washed with PBS and cells were incubated with HFM as described above. After 24 h 10 μM goserelin was added and 12 h later the flasks were irradiated with 4 Gy. 12 h later the cells were detached with 0.5 g trypsin as previously described [20]. The pellets were washed twice with PBS and resuspended with CelLytic buffer (Sigma) containing protease inhibitors (Sigma). Equal amounts of protein per sample were used and diluted to equal volumes with Laemmli buffer. The cell lysates were separated on SDS-PAGE (15%, ProSieve 50 Gel Solution, Cambrex, Verviers, Belgium) under reducing conditions and transferred to nitrocellulose membranes (HybonD-ECL, GE Healthcare Europe, Munich, Germany). The nitrocellulose membranes were blocked with 5% instant skimmed milk powder, spray-dried (Naturaflor, Dietmannsried, Germany) in TBST [137 mmol/L NaCl, 2.7 mmol/L KCl, 0.1% Tween 20, 25 mmol/L Tris-HCl (pH 7.4)] for 1 h at room temperature, washed with TBST, and then incubated at 4°C overnight with rabbit anti-human polyclonal antibodies: anti-c-Fos: (Abcam, Cambridge, UK, nr. 7963 [dilution 1:200 in TBST]), anti-phospho ERK1/2 (Abcam, nr. 9101 [1:1000]), anti-phospho p38 (Abcam, nr. 9211 [1:1000]), and anti-Actin (Sigma, nr. A5060 [1:500]). Following washing the membranes were incubated at room temperature with horseradish peroxidase-conjugated anti-rabbit IgG (GE Healthcare Europe) at a 1:10,000 dilution in TBST for 1 h. After washings, specifically bound antibody was detected using the enhanced chemiluminescence kit (Millipore, Schwalbach, Germany). The bands were analyzed using the Kodak 1D image system (Kodak, New Haven, CT).

### Statistical analysis

For descriptive statistics, the software package KaleidaGraph 3.5 (Synergy Software, Reading, USA) was used. Means and standard deviations were calculated for each of the data points; statistical comparison of the survival data was done using the t-test and one-way ANOVA (Tukey HSD for post hoc testing). P < 0.05 was considered statistically significant. Survival curves, each referring to its specific control, were fitted to the data using the linear-quadratic model. The results of the viability assays were fitted with exponential functions, as this reflects best the biological behaviour of exponentially growing cells.

## Results

### Staining of LHRH-receptors

**Figure 1:**
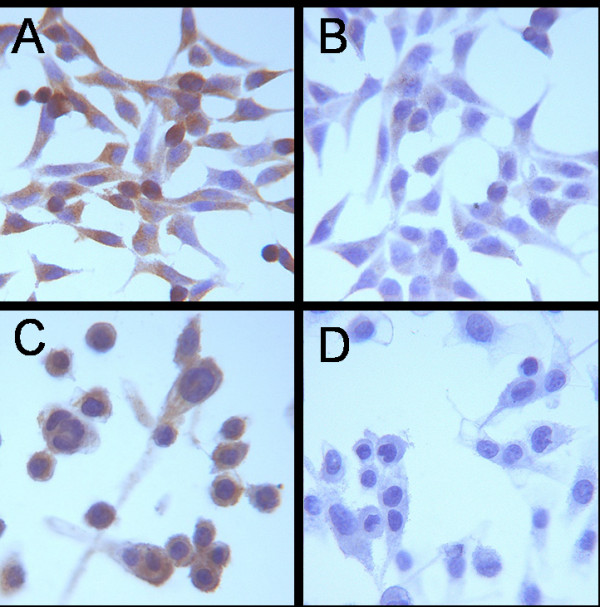
**Immunocytological staining of LHRH in LNCaP (A; B without primary antibody) and PC-3 (C; D without primary antibody)**. Expression of the receptors reflects as a brown staining. Both studied cell lines LNCaP und PC-3 showed expression of LHRH-receptors.

The studied passages of both cell lines LNCaP und PC-3 showed a high expression of LHRH receptors. In figure 1 receptor expression is shown by a brown staining.

### Changes in protein expression

**Figure 2:**
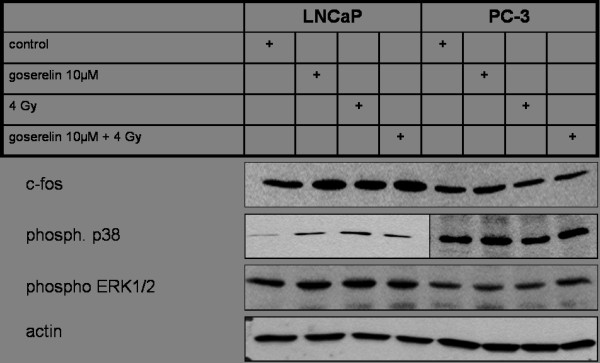
**Western Blot of c-fos, phosphorylated p38 and phosphorylated ERK1/2 after incubation with goserelin and irradiation with 4 Gy**. Cells were incubated with HFM for 24 h, than 10 μM goserelin was added. 12 h later the flasks were irradiated with 4 Gy and after 12 h the cells were trypsinized. In LNCaP incubation with goserelin induced the expression of c-fos more than irradiation alone. In PC-3 the expression of c-fos was not changed. Phosphorylated p38 was induced by goserelin incubation and irradiation in LNCaP. In PC-3, there was a higher expression of this protein after incubation with goserelin than after irradiation alone. Phosphorylated ERK1/2 was induced after goserelin incubation and irradiation in LNCaP, but not in PC-3. These results show, that the incubation with goserelin was functionally active in the tested cell systems.

To test the functional activity of goserelin in the investigated cell lines, we analyzed protein expression and phosphorylation that are involved in functional signalling downstream the LHRH receptor. These include p38, ERK 1/2 and c-fos [21], the results are shown in figure 2.

In LNCaP cells 24 h incubation with 10 μM goserelin or irradiation with 4 Gy induced the expression of c-fos, but the combination of both treatments did not further increase the expression. In contrast, in PC-3 the expression of c-fos was not increased by goserelin incubation and seemed to be slightly reduced by irradiation (when compared to control).

Phosphorylated p38 was induced by goserelin incubation and irradiation in LNCaP cells. In PC-3 cells, we found more expression of this protein after incubation with goserelin than after irradiation alone.

Goserelin incubation and irradiation induced the expression of phosphorylated ERK1/2 in LNCaP cells. In PC-3, only the combination of irradiation and goserelin increased the level of phosphorylated ERK1/2.

These results demonstrate that the incubation with goserelin led to alterations on the protein level. Goserelin incubation was functionally active in our cell systems.

### Cell viability

#### LNCaP

**Figure 3:**
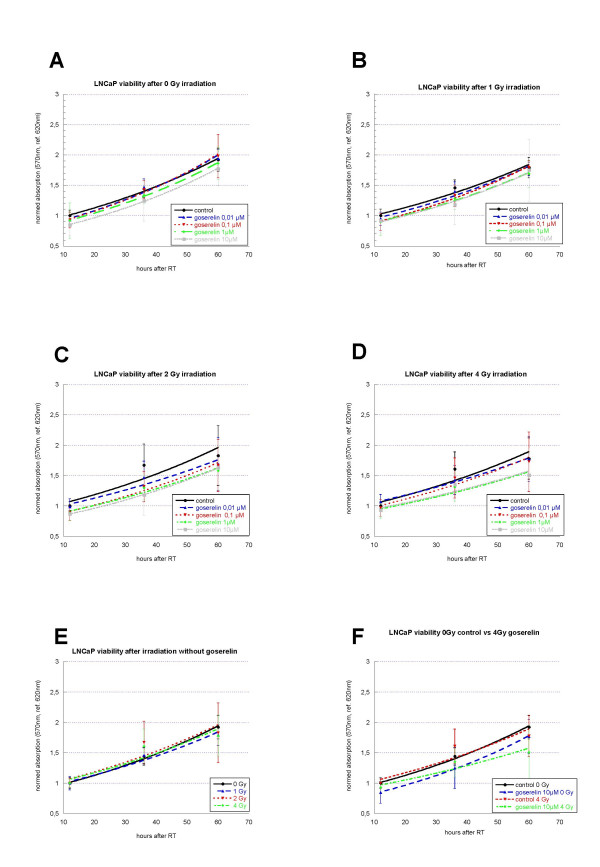
**Viability of LNCaP cells 12 h, 36 h, and 60 h after irradiation (t = 0 h) with 0 Gy (A), 1 Gy (B), 2 Gy (C) and 4 Gy (D) and incubation with different concentrations of goserelin (from 12 h before irradiation on). **Experiments were performed in triplicate and repeated at least three times. At the different time-points the cells were incubated with CTB-reagent for 4 h, than absorption was measured at 570 nm and 620 nm. After blank-reduction radiation the results were normalized to the specific irradiation controls (without goserelin). Results are expressed as measured absorbance. Error bars represent standard deviations. The effect of irradiation alone is shown in figure E. Incubation with goserelin had no significant influence on cell viability. When comparing 0 Gy goserelin control with 4 Gy and 10 μM goserelin only a trend of reduced cell viability is detectable without statistical significance (F; p = 0.23).

During the observation time (up to 60 h after irradiation) the cells showed an exponential growth as expected (figure 3). Interestingly, the incubation with goserelin had no significant influence on cell viability. Furthermore, additional irradiation showed no reduction of cell viability when compared to irradiated controls alone. When comparing 0 Gy goserelin control with 4 Gy and 10 μM goserelin only a trend of reduced cell viability was detectable without statistical significance (figure 3-F, p = 0.23).

Noticeable, irradiation without goserelin proved to be of minor activity regarding cell viability (figure 3-E). This observation reflects the moderate induction of apoptosis by irradiation in this cell line (see discussion) leading to an insignificant reduction in the number of viable cells.

#### PC-3

**Figure 4:**
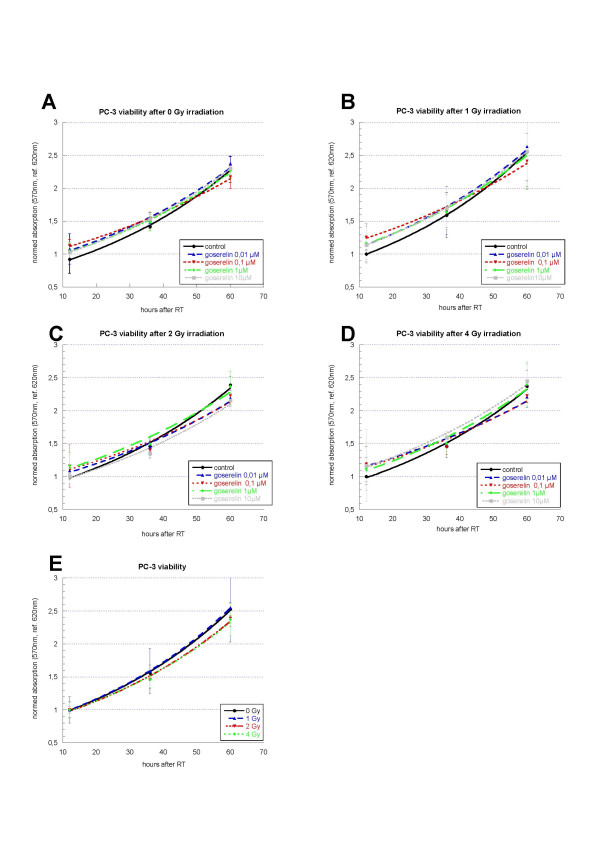
**Viability of PC-3 cells 12 h, 36 h, and 60 h after irradiation (t = 0 h) with 0 Gy (A), 1 Gy (B), 2 Gy (C) and 4 Gy (D) and incubation with different concentrations of goserelin (from 12 h before irradiation on). **Experiments were performed in triplicate and repeated at least three times. At the different time-points the cells were incubated with CTB-reagent for 4 h, than absorption was measured at 570 nm and 620 nm. After blank-reduction radiation the results were normalized to the specific irradiation controls (without goserelin). Results are expressed as measured absorbance. Error bars represent standard deviations. The effect of irradiation alone is shown in figure E. In PC-3, neither incubation with goserelin nor the combination of goserelin and irradiation showed a significant reduction in cell viability when compared to the particular controls.

Also in PC-3 cells an exponential growth kinetic was expected and observed (figure 4). Like in LNCaP, neither incubation with goserelin nor the combination of goserelin and irradiation showed a significant reduction in cell viability when compared to the particular controls.

### Clonogenic survival

#### LNCaP

**Figure 5:**
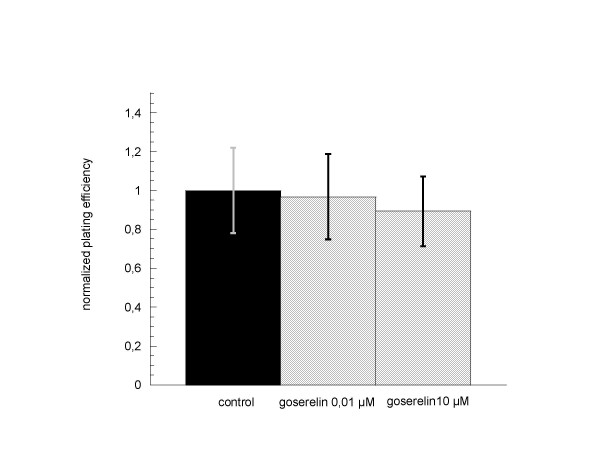
**Clonogenic survival of LNCaP cells after 24 h incubation with goserelin 0.01 μM and 10 μM**. Colonies were evaluated after 6 cell doublings (only colonies > 50 cells counted). Survival was expressed relative to untreated controls. Error bars represent standard errors. There was no significant influence of 24 h goserelin incubation during hormonal withdrawal on clonogenic survival of LNCaP cells. A concentration dependent effect was not seen.

Incubation of LNCaP cells with goserelin in both tested concentrations for 24 h during hormone withdrawal did not show significant influence on clonogenic survival (figure 5).

**Figure 6:**
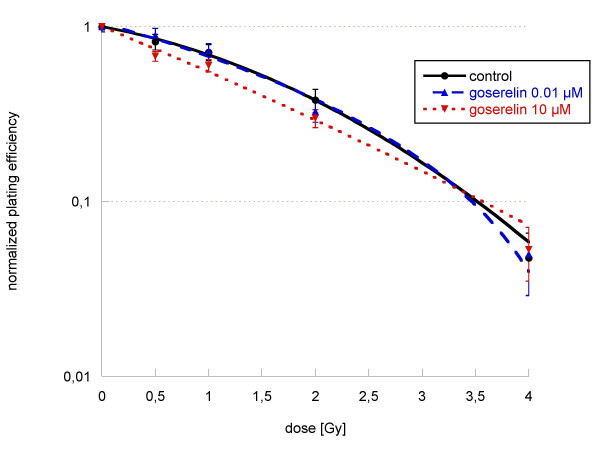
**Clonogenic survival of LNCaP cells after incubation with goserelin 0.01 μM and 10 μM 12 h before and after irradiation with single doses between 0 and 4 Gy**. Colonies were evaluated after 6 cell doublings (only colonies > 50 cells counted). Survival was expressed relative to sham-irradiated controls. Experiments were performed in quadruplicate and repeated at least three times. Error bars represent standard errors. Linear-quadratic equation was used for the control and the goserelin 10 μM curve, a polynominal equation for the goserelin 0.01 μM curve. Incubation with hormones in different concentrations (dotted lines) did not alter cell survival significantly when compared to untreated controls.

Irradiation with 4 Gy alone reduced clonogenic survival under 10% (figure 6). Additional incubation with goserelin 12 h before and 12 h after irradiation did not further decrease clonogenic survival. In this experimental setting goserelin did not show any effects on the clonogenity of LNCaP-cells.

#### PC-3

**Figure 7:**
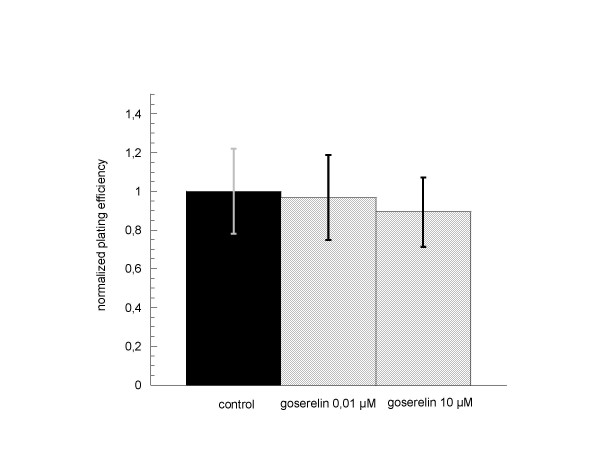
**Clonogenic survival of PC-3 cells after 24 h incubation with goserelin 0.01 μM and 10 μM**. Colonies were evaluated after 6 cell doublings (only colonies > 50 cells counted). Survival was expressed relative to untreated controls. Error bars represent standard errors. There was no significant influence of 24 h goserelin incubation during hormonal withdrawal on clonogenic survival of PC-3 cells. A concentration dependent effect was not seen.

**Figure 8:**
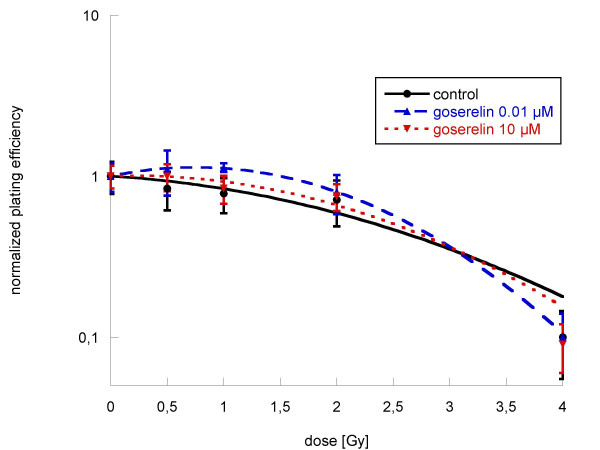
**Clonogenic survival of PC-3 cells after incubation with goserelin 0.01 μM and 10 μM 12 h before and after irradiation with single doses between 0 and 4 Gy**. Colonies were evaluated after 6 cell doublings (only colonies > 50 cells counted). Survival was expressed relative to sham-irradiated controls. Experiments were performed in quadruplicate and repeated at least three times. Linear-quadratic equation was used for all curves. Error bars represent standard errors. Incubation with hormones in different concentrations (dotted lines) did not alter cell survival significantly when compared to untreated controls.

The same results were obtained in PC-3 cells. Neither incubation with goserelin alone in different concentrations (figure 7) nor additional incubation with goserelin in combination with radiation therapy showed any significant influence on clonogenic cell survival when compared to the particular controls (figure 8).

## Discussion

We investigated the influence of incubation with goserelin on the radiosensitivity of PC cells *in vitro*. This is of particular interest, as about 85% of PC express LHRH-receptors [15]. Using immunohistochemistry we detected LHRH expression in our cell lines, and in Western Blot analysis we could show an effect of goserelin incubation on protein expression independent of irradiation effects.

Our experiments were done under androgen deprivation (AD) (medium supplemented with charcoal-stripped fetal bovine serum) to mimic the clinical situation. Goserelin exposure of PC patients causes AD after about two weeks. Under our experimental conditions we could not demonstrate any significant influence of goserelin on radiosensitivity of the tested PC cell lines. Therefore, our working hypothesis was disproved: direct interaction of goserelin with PC cells during irradiation does not seem to explain the clinically observed increase of overall survival in patients after combined therapy.

Recent studies investigated the effects of AD and radiotherapy in PC cell lines *in vitro *[summarized in 9]. In LNCaP cells AD led to growth delay. This delay could be abolished by incubation with synthetic androgens. No supraadditive effects on clonogenic survival were observed, when AD was combined with irradiation. In these experimental settings androgens were withdrawn in varying protocols 3 to 5 days before irradiation. After irradiation the cells were subjected to "immediate plating" for methodological reasons. This means that the monolayer had to be trypsinized, counted and seated before colony formation took place. In contrast, we tested much shorter goserelin incubation times. This allowed us to investigate the direct interaction between goserelin and radiotherapy. We were not interested in long-term exposure of goserelin before or after irradiation. Furthermore, as we avoided "immediate plating", we could exclude possible errors caused by this methodology.

Other studies investigated the interaction between AD and radiotherapy *in vivo*. Subcutaneous tumors were grown and AD was performed by means of surgical castration in male mice. Several studies demonstrated a significant reduction of the TCD50 by the combination therapy depending on the timing of AD [10-13].

Taken together in vivo and in vitro data support the hypothesis, that androgen withdrawal during irradiation increases clonogenic cell death in an additive manner [9]. Furthermore, the combination therapy leads to reduced growth kinetics after irradiation. Both factors together may explain the clinically observed survival benefit [9].

We chose to test goserelin concentrations between 0.01 – 10 μM. These concentrations showed significant effects on proliferation of PC-cells, receptor binding and other endpoints in vitro [22,23]. In patients serum concentrations of about 2 μM are reached after implantation of a goserelin depot, with renal insufficiency up to 10 μM [24].

One proposed mechanism of biological activity of LHRH in prostate carcinoma is that LHRH signalling involves MAPK-kinases. Among others, LHRH receptors trigger PLC to activate PKC. PKC activation limits EGFR tyrosine kinase activity by phophorylating EGFR at threonine 654 [25].

We studied the effects of goserelin incubation on two main MAPK-pathways (phos. p38 and phosphor ERK1/2) and on c-fos 24 h after start of incubation, 12 h after irradiation (to reflect long term effects). In this setting, we could demonstrate biological effects of goserelin on protein expression and phosphorylation. Goserelin incubation was functionally active in our cell systems.

The observation, that the incubation with goserelin induced the c-fos expression in LNCaP cells is in contrast to other reports. Dondi et al. incubated LNCaP cells with 10 μM LHRH agonist for 30 or 60 min, than added EGF to the media during the last 30 min of incubation [26]. The expression of c-fos was determined by Northern blot analysis. Under these conditions the LHRH agonist completely abrogated the EGF induced stimulation of c-fos mRNA. On the other hand, in several human endometrial, ovarian and breast cancer cell lines an incubation of quiescent cells for about 30 min with 1 μM triptorelin did not alter c-fos mRNA expression in semiquantitative RT-PCR [27]. These observations are in line with our results in PC-3 cells. However, it is difficult to compare the results of these studies, because they tested different incubation times of LHRH analoga, investigated different cell systems and analyzed different endpoints (e.g. RNA-expression vs. protein expression).

Noticeable was the minor influence of irradiation and the combination of goserelin incubation and irradiation on cell viability. One limitation of this method was the measurement of exponentially growing cultures. Minor inaccuracies in cell plating at the start of each assay aggravated over time, resulting in substantial differences in cell numbers at the time of measuring cell viability. This led to standard deviations of up to 40%. A further obstacle was, that metabolic activity assays seldom reflect cell cytotoxicity from RT in solid malignancies. We used this assay to investigate for growth arrest and apoptosis induced by the two treatment modalities. However, directly after irradiation cell numbers will not be reduced in these solid tumor cell lines. The induction of apoptosis has been investigated in LNCaP and PC-3 cells grown in standard medium by several groups. With different methodological approaches only a minor induction of apoptosis was seen 24 h after irradiation with 4 – 5 Gy (< 5% more than in sham-irradiated controls) [28-30]. One study showed increased rates of apoptosis after irradiation of cells grown in charcoal-stripped serum, but could not demonstrate any dose dependence [9]. In fractionated experiments AD did no further increase induction of apoptosis [12]. The authors concluded that apoptosis did not play a major part in tumor control in this experimental design.

Taken together, our cell viability data are easily explained by published results of other studies.

## Conclusion

We could not demonstrate any significant effects of goserelin on irradiated PC cell lines *in vitro*. The clinically observed increase in tumor control after combination of goserelin with radiotherapy in PC cannot be attributed to an increase in radiosensitivity of PC cells by goserelin *in vitro*. It is to be explained by the synergistic effects of AD and irradiation.

Although studies on the combinatory effects of LHRH receptor-**antagonists **(e.g. cetrurelix) and radiotherapy are still missing, we suppose no detrimental effects on tumor control, as the incubation with LHRH receptor-**agonists **proved not to increase radiosensitivity of PC cell lines.

## Competing interests

Potential conflicts of interest exist: Astra-Zeneca granted 20 mg of goserelin to support our study (value about 500,- €). R.M.H. received honoraria for lecturer activities by Astra-Zeneca under 1000,- €. The authors assure that the acquisition and interpretation of laboratory data were not influenced by the donation and the honoraria.

## Authors' contributions

RMH designed the study, coordinated the work and drafted the manuscript

DS did the cytological work and helped with irradiation tests

SF and CG designed the functional studies and performed receptor analysis and the Western blots

MRF coordinated the work, interpreted the data and helped drafting the manuscript

MN and AH did the irradiations and performed statistical analysis

PT and HS participated in the study design and interpretation of the data

HC conceived the study, and participated in its design and coordination and helped to draft the manuscript

All authors read and approved the final manuscript.
